# The usefulness of SAGE score in predicting high pulse wave velocity in hypertensive patients: a retrospective cohort study

**DOI:** 10.3389/fcvm.2024.1227906

**Published:** 2024-03-26

**Authors:** Luiz Carlos Carneiro Pereira, Patrícia Chagas, Eduardo Costa Duarte Barbosa, Weimar Kunz Sebba Barroso, Adriana Camargo Oliveira, Suélen Feijó Hillesheim, Vitória Carolina Kohlrausch, Diego Chemello

**Affiliations:** ^1^ Postgraduate Program in Gerontology, Universidade Federal de Santa Maria (UFSM), Santa Maria, Brazil; ^2^ Department of Food and Nutrition, Universidade Federal de Santa Maria (UFSM), Santa Maria, Brazil; ^3^ Department of Cardiology, Complexo Hospitalar Santa Casa de Misericórdia de Porto Alegre – Cardiologia, Porto Alegre, Brazil; ^4^ Department of Cardiology, Universidade Federal de Goiás – Liga de Hipertensão Arterial, Goiânia, Brazil; ^5^ Faculty of Medicine, Universidade Federal de Santa Maria (UFSM), Santa Maria, Brazil

**Keywords:** hypertension, vascular stiffness, pulse wave velocity, risk prediction, blood pressure

## Abstract

**Introduction:**

Aortic stiffness assessed by pulse wave velocity (PWV) is an important predictor to evaluate the risk of hypertensive patients. However, it is underutilized in clinical practice. We aimed to identify the optimal cutoff SAGE score that would indicate a risk PWV ≥ 10 m/s in Brazilian ambulatory hypertensive patients.

**Materials and methods:**

A retrospective cohort study. Patients underwent central blood pressure measurement using a validated oscillometric device from August 2020 to December 2021. A ROC curve was constructed using the Youden statistic to define the best score to identify those at high risk for PWV ≥ 10 m/s.

**Results:**

A total of 212 hypertensive individuals were selected. The mean age was 64.0 ± 12.4 years and 57.5% were female. The following comorbidities were present: overweight (47.6%), obesity (34.3%), and diabetes (25.0%). Most of the sample (68.9%) had PWV < 10 m/s. According to Youden's statistic, a cutoff point of 6 provided the optimal combination of sensitivity and specificity for identifying patients with a PWV ≥ 10 m/s. This cutoff achieved sensitivity of 97.0%, and specificity of 82.9%. In clinical practice, however, a cutoff point of 7 (where score values of at least 7 were considered to indicate high risk) had a positive likelihood ratio of 8.2 and a negative likelihood ration of 0.346, making this the ideal choice by accurately excluding patients who are less likely to have PWV ≥ 10 m/s.

**Conclusion:**

A SAGE score ≥7 identified Brazilian hypertensive patients with a high risk of PWV ≥ 10 m/s.

## Introduction

Pulse wave velocity (PWV) is an important tool for the early identification of vascular damage caused by elevated blood pressure (BP), or the presence of other associated factors with accelerated vascular aging (1, 2). The use of PWV as a biomarker that can gauge the overall risk of patients, identify organ damage, and facilitate clinical decision-making has been acknowledged by guidelines and consensus documents mainly, but nonexclusively for hypertensive patients (3–5).

Carotid-femoral PWV is considered the gold-standard method for arterial stiffness, and it´s been used mainly in western countries (6). However, other methods for PWV measurement have been validated, like brachial-ankle PWV (7). Over the last years, some devices claim to estimate PWV from a single brachial cuff pressure recording, like the Cardio Mapa AOP® (Cardios, São Paulo, Brazil). By this method, central systolic BP was calculated using the ARCSolver® (Austrian Institute of Technology, Vienna, Austria) algorithm, which determines the aortic systolic BP. The aortic systolic BP can be calculated by the algorithm by two different calibration methods: C1 (using brachial systolic and diastolic BP), and C2 (using oscillometrically measured mean/diastolic BP) (8).

Despite growing evidence for the clinical applicability of noninvasive measurement of PWV (4, 9–10), its implementation in clinical practice is suboptimal and restricted to tertiary and research centers. This can be attributed to lack of regulation and reimbursement from healthcare authorities and cost of dedicated devices, among other factors (11).

The SAGE score is based on four clinical parameters (peripheral systolic blood pressure, age, fasting glucose, and glomerular filtration rate calculated by CKD-EPI) (11). It has been validated in European and Japanese populations, as well as in a Brazilian population (11–13). It has been used to screen and identify hypertensive patients with an elevated likelihood of PWV and a resulting high risk of cardiovascular events. Despite these important validation studies in hypertensive individuals, continuous efforts to validate the SAGE score throughout different communities have been made, particularly those with poor access to PWV analysis methods (12).

As such, the present study aimed to identify a SAGE score that would indicate a high risk of PWV ≥ 10 m/s in Brazilian hypertensive patients who had their PWV measured by an oscillometric device.

## Materials and methods

This retrospective study included medical records of outpatients who consulted in a private cardiology center in Brazil. We conducted a retrospective analysis of patients who had undergone central blood pressure measurement (CBPM) using the oscillometric method from August 2020 to December 2021. The present study was approved by the Research Ethics Committee of the Federal University of Santa Maria (UFSM), RS, Brazil (CAAE 51438421.4.0000.5346) and conducted according to the Declaration of Helsinki. We included patients with 18-years-old or older with the diagnosis of systemic arterial hypertension (SAH) who consulted in the referred service. Hypertensive patients were defined as those who had high blood pressure at the doctor's office, a CBPM of ≥140/90 mmHg, or an overall mean ≥130/80 mmHg in ambulatory blood pressure monitoring (ABPM) or were using antihypertensive medications (11). The glomerular filtration rate (GFR) was estimated using the creatinine value using the Chronic Kidney Disease Epidemiology Collaboration (CKD-EPI) equation.

### Measurement of pulse wave velocity

The parameters central systolic blood pressure (SBP), central diastolic BP (DBP), peripheral SBP, peripheral DBP, PWV, and augmentation index (Aix) were obtained using a validated oscillometric device, the Dyna Mapa AOP® (Cardios, São Paulo, Brazil) (14, 15), based on triplicate measurements of PWV with C2 calibration (diastolic mean), and the data were processed with the ARCSolver® algorithm (Austrian Institute of Technology, Vienna, Austria). The measurements were performed on the left arm, with the patient in a seated position, with the legs uncrossed, feet flat on the floor, and the arm resting at heart level on a table. Patients were instructed to avoid alcohol consumption for 10 h and refrain from caffeine intake, smoking, and exercise for 3 h immediately prior to the measurement and to rest for 10 min before the procedure (16). Three readings of the central blood pressure values were obtained, and the average of the three measurements was calculated.

### Calculation of the SAGE score

SAGE is the English acronym used to define the score variables: SBP, age, glucose, and estimated GFR. Each component of the acronym was categorized, and each category received a score; the SAGE score received a score from 0 to 17 points (11). After the SAGE calculation, the overall sample of hypertensive patients and those with PWV ≥ 10 m/s were divided into score categories from 0 to 17 to analyze the frequency of the scores. PWV values ≥10 m/s are related to increased aortic stiffness in hypertensive patients and the presence of target organ lesions (11).

### Statistical analysis

The analyses were performed with the Statistical Package for Social Sciences (SPSS), version 21.0. The distribution of quantitative data was verified using the Kolmogorov–Smirnov test. The continuous variables were described as mean and standard deviation, or median and interquartile range, according to the distribution of data. Categorical variables were presented as absolute and relative values.

For each SAGE score from 0 to 17, analysis of sensitivity (SE), specificity (SP), positive predictive value (PPV), and negative predictive value (NPV) for PWV ≥ 10 m/s was performed, and a receiver operating characteristic (ROC) curve was constructed. The optimal cutoff point for the SAGE score to identify patients at high risk for high PWV was chosen using the Youden J index. ROC curve >0.7 was considered to indicate sufficient predictive accuracy. The cutoff point for the SAGE score was established using three criteria: higher Youden Index, sensitivity of at least 0.80 and specificity of at least 0.60. The analyses with *P* < 0.05 were considered significant.

In addition to the statistical analysis obtained by the ROC curve graph, the cutoff point was also analyzed using a qualitative approach to determine the ideal cutoff point (17).

## Results

**Figure 1:**
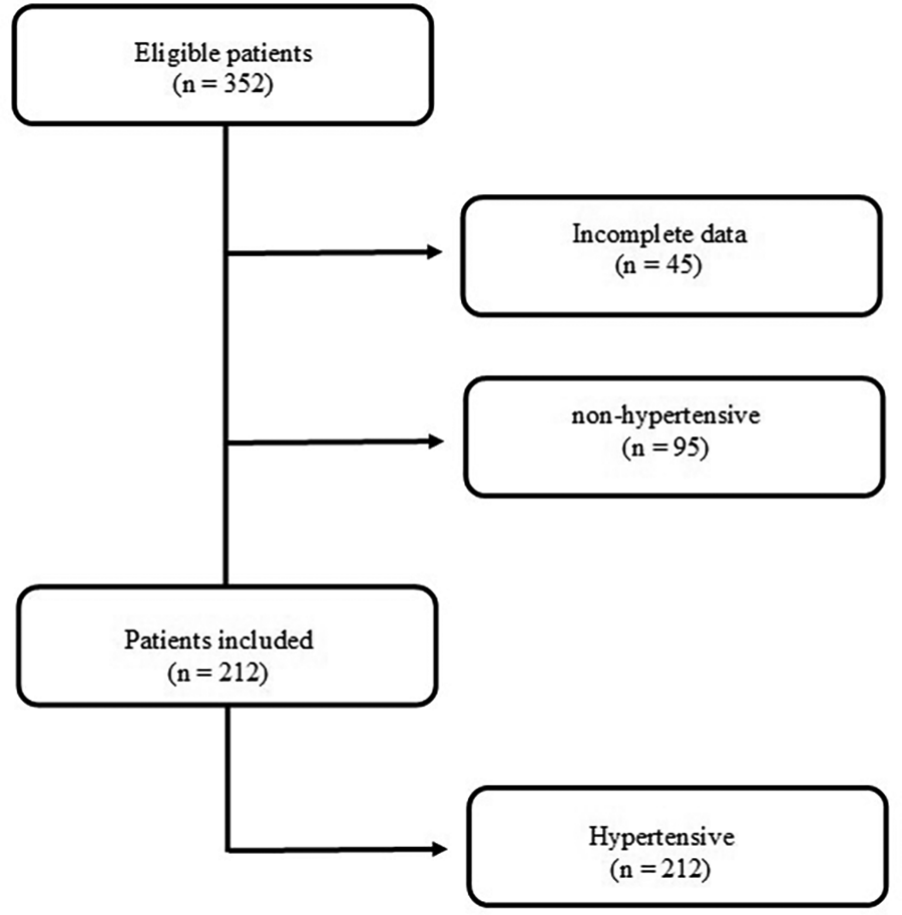
Flowchart for selection of participants. A total of 212 patients were included (diagnosed with systemic arterial hypertension).

A total of 352 patient who underwent CHPM were identified. Of these, 212 were selected. Forty-five patients were excluded due to absence of clinical data necessary to calculate the SAGE score, and 95 because they were non-hypertensive (Figure 1).

**Figure 2:**
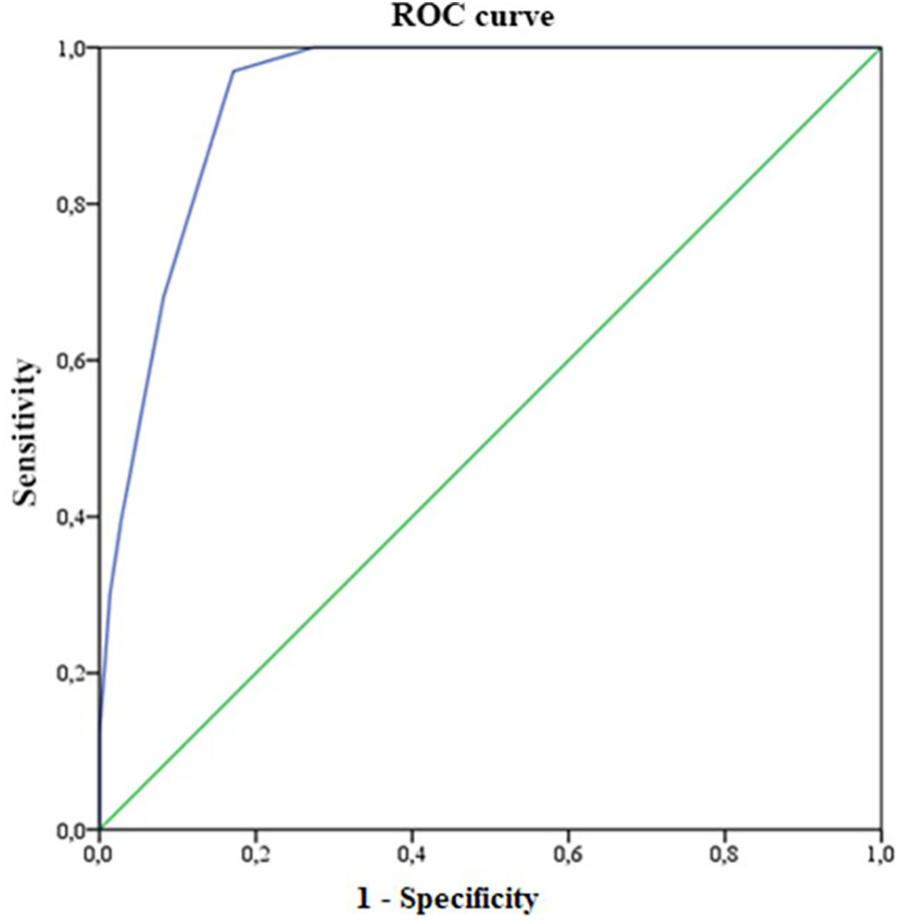
ROC curve for measuring the SAGE score in predicting cardiovascular risk (PWV ≥ 10 m/s) in hypertensive patients (*n* = 212).

The mean age of the sample was 64.0 ± 12.4 years (range 30–89 years), most often female (57.5%), overweight (47.6%) or obesity (34.3%), non-diabetic (75%) (Table 1). Most had PWV values < 10 m/s (68.9%). The performance of the SAGE score in predicting elevated PWV was analyzed. The sensitivity and specificity of different cutoff points are shown in Table 2. For the 212 patients, in the ROC analysis, the area under the curve (AUC) was 93.8% (95% CI from 90.8% to 96.8%, *P* ≤ 0.001) (Figure 2).

**Table 1:** Sociodemographic, anthropometric, and clinical characteristics of hypertensive patients seen in a private cardiology service in the city of Santa Maria, Brazil.

Features	*N* = 212
Sociodemographic	
Age (years)	64.0 ± 12.4
Sex	
Female	122 (57.5)
Male	90 (42.5)
Anthropometric	
Weight (kg)	79.0 ± 16.4
Height (cm)	163.1 ± 21.5
Body mass index (kg/m^2^)	28.7 ± 4.6
Nutritional status (BMI)	
Low weight	2 (1.0)
Eutrophic	36 (17.1)
Overweight	100 (47.6)
Obesity	72 (34.3)
Clinics	
Peripheral systolic blood pressure (SBP) (mmHg)	130.1 ± 17.5
Peripheral diastolic blood pressure (DBP) (mmHg)	82.0 ± 12.3
Central SBP (mmHg)	116.6 ± 13.9
Central DBP (mmHg)	82.8 ± 11.9
Augmentation index (Aix)	24.8 ± 10.2
Pulse wave velocity (PWV) (m/s)	9.2 ± 1.9
PWV	
<10 m/s	146 (68.9)
≥10 m/s	66 (31.1)
Fasting plasma glucose (mg/dl)	102.0 ± 22.7
Diabetes mellitus	
No	159 (75.0)
Yes	53 (25.0)
Glomerular filtration rate (ml/min/1.73 m^2^)	88.9 ± 34.4
Creatinine	0.9 ± 0.2
SAGE score (median and interquartile range)	5.5 (3.3–8)

Quantitative variables with normal distribution are described as mean and standard deviation; the nonparametric variable (SAGE score), is describes in the form of median and interquartile range. Categorical variables in the form of absolute and relative values. The missing data were: one for weight; two for central SBP, and central DBP, Aix; three for height, BMI, and nutritional classification.

**Table 2:** Detailed report for the sensitivity and specificity of different cut points of the SAGE score in patients with hypertension from a private cardiology service in the city of Santa maria, Brazil (*N* = 212).

Cut point	Sensitivity (%)	Specificity (%)	Correctly classified (%)	+Likelihood ratio	−Likelihood ratio
>0	100	11	33.7	1.1231	0.0000
>1	100	11	33.7	1.1231	0.0000
>2	100	26	37.9	1.3519	0.0000
>3	100	36	41.5	1.5699	0.0000
>4	100	49	47.1	1.9730	0.0000
>5	100	73	62.3	3.6500	0.0000
>6	97	83	71.9	5.6630	0.0366
>7	68	92	78.9	8.2955	0.3467
>8	39	97	86.7	14.3788	0.6231
>9	30	99	90.9	22.1212	0.7066
>10	21	99	93.3	30.9697	0.7933
>11	12	100	100.0		0.8788
>12	08	100	100.0		0.9242
>13	03	100	100.0		0.9697
>14	02	100	100.0		0.9848

According to Youden's J statistic, a cutoff point of 6 provided the optimal combination of sensitivity and specificity for identifying patients with a PWV ≥ 10 m/s in individuals with SAH. Table 2 shows the ability of this cutoff point in hypertensive subjects. The values were as follows: SE of 97.0%, SP of 82.9%, PPV of 71.9%, and NPV of 98.4%. For this cutoff point, a positive test is about 5.6630 times more likely to be obtained in the presence of the disease than in the absence of it. If the test with the SAGE score is negative, the likelihood ratio is 0.0366.

Despite the Youden's J statistic demonstrated the cutoff point of 6 as the optimal combination of sensitivity and specificity for identifying patients with a PWV ≥ 10 m/s, the choice of a cutoff point of 7 improved the specificity, at the expense of sensitivity. A cutoff point of 7 (where score values of at least 7 were considered to indicate high risk) had a positive likelihood ratio of 8.2 and a negative likelihood ration of 0.346. Thus, the use of this cutoff point would aid decision-making by accurately excluding patients who are less likely to have PWV ≥ 10 m/s.

## Discussion

In this cross-sectional study, we reported the SAGE cutoff point to identify increased PWV using a validated oscillometric device in a Brazilian population of 212 hypertensive patients. Using the quantitative approach (based on the Youden index), the cutoff point was 6. However, using a qualitative approach that prioritized achieving satisfactory PPV while maintaining a high NPV, a SAGE cutoff of 7 was chosen as the best option. With this cutoff point, its emphasized that patients not selected for PWV measurement would have a low probability of PWV ≥ 10 m/s. This strategy optimizes financial resources in places with health systems that have limited PWV analysis availability (11, 13).

Our findings are similar to those reported by Tomiyama et al., who defined a SAGE cutoff point of 7 for Japanese hypertensive patients undergoing brachial-ankle PWV measurement (13). However, we reported slightly different cutoff than the one reported by Xaplanteris et al. and Oliveira et al. (11, 12). In 2019, Xaplanteris et al. validated the SAGE score using tonometry in a Greek population of patients with SAH (11). They defined the SAGE score cutoff of 8 as the best predictor of high PWV. More recently, Oliveira et al. identified the same SAGE score of ≥8 for predicting high PWV in a population of Brazilian hypertensive patients (12). In the last study, the authors measured PWV with the same oscillometric technique described in our study (12, 14, 15). The distinct cutoff observed in these studies could be related to methodological differences used to calculate the SAGE score and to measure PWV, particularly in the study by Tomiyama et al. in the last study (12, 18, 19). In the present study, the estimation of PWV was based on the Dyna Mapa AOP® oscillometric device based on its advantages and accessibility in our community (12, 14, 15). Besides it, there is a series of longitudinal studies showing a good correlation with target organ lesions and cardiovascular events with oscillometric devices (20–22), when compared to the gold standard noninvasive method of carotid-femoral tonometry (15). The differences observed between our data and the study by Oliveira et al. (12) could be related, at least partially, by regional and ethnical variations in the Brazilian population (23, 24). Additionally, the central systolic BP differences observed between C1 and C2 calibrations must also been acknowledged. Like Oliveira et al., we used C2 calibration (12). Regarding clinical validation, studies have focused on central systolic BP whereby C2 calibration is superior to cuff brachial SBP and C1 calibration in terms of association with organ damage (25–27) and mortality outcomes (27).

The present study reinforces the importance of optimizing PWV measurement in clinical practice of patients with SAH, because this technique is still restricted to tertiary centers (2, 11). In this setting, the SAGE score becomes a simple clinical tool to identify those patients who should undergo PWV measurement. Like Oliveira et al. (12), our paper evaluated the SAGE score cutoffs against oscillometric measurements in Brazilian hypertensive patients. The present study has some limitations. First, the SAGE cutoff was obtained using data from a specific Brazilian population in south of Brazil, with mixed ethnicity (24). The sample size was small, with different ethnic background compared to the previous studies. Reference values for PWV have been defined in the Brazilian population for categories defined by age, sex, and cardiovascular risk factors (28). However, the present study defined abnormal PWV as values greater or equal than 10 m/s, according to the original validation of the SAGE score (11).

Regarding future clinical implications, we believe that further studies with larger sample size that involves most Brazilian regions and the application of SAGE score in non-hypertensive individuals will be useful for determining the use of this score.

## Conclusion

The SAGE score presented a good performance as a predictor of PWV measured in Brazilian hypertensive outpatients, using oscillometric device. The cutoff point was the same as reported in the Japanese cohort and close to that reported in the European cohort and the first published Brazilian cohort. Our data reinforce that the SAGE score is a useful and robust tool for identification of hypertensive individuals with probable PWV ≥ 10 m/s.

## Data Availability

The raw data supporting the conclusions of this article will be made available by the authors, without undue reservation.
